# Image Encryption Algorithm Based on a Novel Hyperchaotic Map and 3D Histogram Model

**DOI:** 10.3390/e28050576

**Published:** 2026-05-21

**Authors:** Xiaoqiang Zhang, Pengfei Chen, Xueheng Zhang

**Affiliations:** School of Information and Control Engineering, China University of Mining and Technology, Xuzhou 221116, China; ts23060127p31@cumt.edu.cn (P.C.); qq1755521127@gmail.com (X.Z.)

**Keywords:** image encryption, chaotic system, simultaneous scrambling–diffusion, 3D histogram

## Abstract

Digital images are easily transmitted in Internet, but there is also a great risk of information leakage. To meet the requirements of secure image transmission and real-time communication, an image encryption algorithm based on a novel chaotic map and a three-dimensional histogram is proposed. Firstly, a novel two-dimensional chaotic map is designed. Compared with traditional chaotic systems, it exhibits superior chaotic performance and a wider parameter range; secondly, the proposed algorithm is designed to extend the original image to three dimensions, followed by 3D simultaneous scrambling–diffusion; thirdly, the 2D exclusive OR (XOR) operation is performed for further diffusion; finally, the 3D matrix is merged to obtain the encrypted image. The encrypted images have uniform histograms and pass the Chi-square test. Information entropy is greater than 7.9992, and the average values of Number of Pixels Change Rate (NPCR) and Uniform Average Change Intensity (UACI), being 99.6137 and 33.4783, respectively, show that this algorithm can effectively resist differential attacks. On average, a 512 × 512 image can be encrypted in 0.7 s using the proposed algorithm. Thus, the proposed algorithm is applicable to image transmission over network platforms due to its high security, excellent encryption performance, and high efficiency.

## 1. Introduction

The widespread use of the Internet has greatly facilitated the transmission of digital images. However, its inherent openness also exposes these images to risks of interception and data leakage during transmission [1]. As a result, enhancing the security of digital images has become a critical concern in the field of network information security [2]. Image encryption serves as a key technique for safeguarding image content. Such encryption algorithms typically introduce confusion and diffusion into the original image, rendering it unrecognizable. Even if an encrypted image is intercepted, an attacker who is unable to break the encryption algorithm can gain no meaningful information from it.

Traditional cryptography algorithms, including the RSA algorithm, Advanced Encryption Standard (AES), Data Encryption Standard (DES), etc., are designed for text information [3]. Theoretically, these encryption algorithms can also be used for digital image encryption. However, there are disadvantages such as low efficiency and a complex calculation process [4], which is due to the characteristics of image data, including large amounts of information, high redundancy, and strong correlation. These traditional algorithms cannot meet the requirements of real-time image encryption. Many image encryption algorithms have been proposed to protect the security of image content. These are based on various techniques, such as compressed sensing [5,6,7], wavelet transform [8,9], S-box [10], chaotic systems [11,12,13], and image recognition [14]. Among them, chaotic systems are widely used for image encryption due to their high computational efficiency [15]. Additionally, Gandelman presents a compact, tabletop emulation of the six-state protocol, implemented with a pulsed laser source and bulk polarization optics to reproduce the essential protocol-level logic and expected basis-dependent statistics of quantum key distribution in a controlled classical setting [16].

Recently, chaotic-systems-based cryptography has become very popular and is applied in different image encryption techniques. Chaotic systems show the behavior of pseudo randomness and possess a number of important features: unpredictable orbital growth, increased sensitivity to initial circumstances and factors, and ease of hardware and software implementation to boost the rate of encryption [17]. Chaotic systems can generally be divided into one-dimensional (1D) chaos and high-dimensional chaos. High dimensional chaotic systems usually have better performance and higher security, but at the same time their structure is complex and the calculation costs are high. In contrast, 1D chaotic mapping is preferred because of its simple structure, easy implementation, and low computational complexity [18]. Zheng et al. constructed a cascade two-dimensional (2D) chaotic map and obtained a good chaos effect, but they did not associate *x* and *y* with each other [19]. In essence, they constructed two independent 1D chaotic systems. Xiong et al. also made the same mistake when constructing high-dimensional chaotic systems [20], and at the same time, they did not make corresponding analysis of the constructed chaotic map and could not show its performance. Wang and his colleagues proposed a modified Chebyshev chaotic map, which offers a more even distribution of chaotic sequences compared to the conventional Chebyshev map [21]. Nevertheless, its chaotic interval still contains numerous periodic windows. Meanwhile, Zhu et al. [22] built a one-dimensional piecewise quadratic polynomial chaotic map by combining the Logistic and Sine maps. Unfortunately, this map fails to remain chaotic when its control parameters are set to small values. Consequently, these one-dimensional chaotic maps suffer from several limitations, including a narrow range of control parameters, poor randomness in the generated pseudo-random sequences, and the existence of periodic windows. Chen et al. proposed a cascade chaotic system based on the Cubic map and Cosine map, which has a larger parameter range and a wider chaotic region [23]. Wang et al. combined the Logistic map with the Sine map and introduced the delayed-state x_i−1_. Chaos x_i+1_ is not only disturbed by the current state variable, but is also affected by the previous state, so the new chaotic map obtains excellent performance [24].

Most image encryption algorithms include the scrambling and diffusion stages [25]. At the scrambling stage, the pixel positions are changed and the correlation of the original pixels is broken. The diffusion stage is required to change the pixel value so as to improve security performance. The two stages of scrambling and diffusion have a clear division of labor, which can usually produce a better result, but the operation complexity is higher. In contrast, the unusual structure of simultaneous scrambling–diffusion can reduce the algorithmic complexity and obtain good results [26].

Zhu et al. adopted the Rubik’s cube model. They divided the original image into 8 × 8 small blocks and transformed the pixels to the bit-level to form several 8th-order Rubik’s cubes [27]. The three-directional (3D) rotation controlled by chaos can fully disturb the Rubik’s cube, and because it is a bit-level operation, it can realize the effect of simultaneous scrambling–diffusion. Chen et al. adopted a DNA-level Rubik’s cube model to reduce the complexity of the simultaneous scrambling–diffusion stage [28]. However, due to the introduction of DNA operation, it is time-consuming in the coding and decoding stages, so the overall efficiency of the algorithm is not ideal. Sheng et al. proposed a simultaneous scrambling–diffusion algorithm based on a Latin square [29]. They used a Latin square to select the concatenation pixel matrix and the key stream matrix and combined them to complete diffusion. This algorithm represents higher innovation and results in a better encryption effect. On the basis of 2D index scrambling, Wen et al. added spiral scanning to change the order, and at the same time changed the pixel value with optional operations such as addition, subtraction, or cyclic shift [30]. The operation was relatively simple and achieved good results.

To ensure the security of images and improve the efficiency of encryption, an image 3D encryption method inspired by the 3D histogram model is proposed in this paper. The main contributions of this paper are highlighted below.

(1) A novel hyperchaotic system is proposed, and a series of performance analyses are conducted to demonstrate its excellent ergodicity and randomness.
(2) A 3D image encryption method is constructed by extending conventional 2D images into a 3D model. The definitions of 3D histogram scrambling and diffusion are presented, and image confusion is achieved via 3D histogram operations.
(3) A 3D histogram simultaneous scrambling–diffusion system is proposed. Based on the designed 3D histogram model and the corresponding scrambling–diffusion operations, an image encryption algorithm based on chaotic maps and the 3D histogram model is proposed.

The remainder of this paper is organized as follows. Section 2 presents the novel chaotic map, analyzes its performance, and provides a brief introduction to the image three-dimensional histogram model. Section 3 details the proposed novel image encryption algorithm. Section 4 describes the experimental setup and results. Section 5 presents a comprehensive analysis of the algorithm. Finally, Section 6 draws conclusions and outlines future research directions.

## 2. Preliminary Work

### 2.1. Chaotic System

Traditional 1D chaotic maps have a simple structure, but suffer from inherent drawbacks such as narrow chaotic parameter ranges, weak randomness of generated sequences, and numerous periodic windows. Most existing 2D chaotic maps fail to achieve strong cross-coupling between state variables; they are essentially two independent 1D chaotic maps spliced together, and can hardly meet the security requirements of high-security image encryption.

This paper selected cosine and arccosine functions to construct the chaotic system based on the dual considerations of practicality and security. The two functions are inverse to each other, so their value ranges are naturally matched during calculation, which avoids problems like invalid iteration and numerical deviation, and ensures the stable operation of the whole system. According to the subsequent chaotic performance analysis, their combination can produce a sufficiently strong nonlinear effect, and the generated random sequence has higher randomness for better encryption security.

On this basis, this paper takes the cosine and arccosine functions as the nonlinear kernel, introduces a bidirectional cross-coupled structure, and proposes a novel 2D Cosine-Arccosine Cross-Combined Map (2D-CACCM). Its mathematical expression is shown in Equation (1):

(1) $$\left\{\begin{array}{l} x_{n+1}=\left|\cos(\pi \times (\arccos(y_{n})+a\times \arccos(x_{n}))\right|\\ y_{n+1}=\left|\cos(\pi \times (\arccos(x_{n})+b\times \arccos(y_{n}))\right| \end{array}\right.,$$

where |·| is absolute value function and *a*, *b* are the control parameters; we set *a*, *b* ∈ (0.1, 100) in this paper. When the initial values of the system are in (0, 1), the sequences generated by 2D-CACCM iteration are evenly distributed in (0, 1).

### 2.2. Chaotic Performance Analysis

#### 2.2.1. Bifurcation Diagram

**Figure 1:**
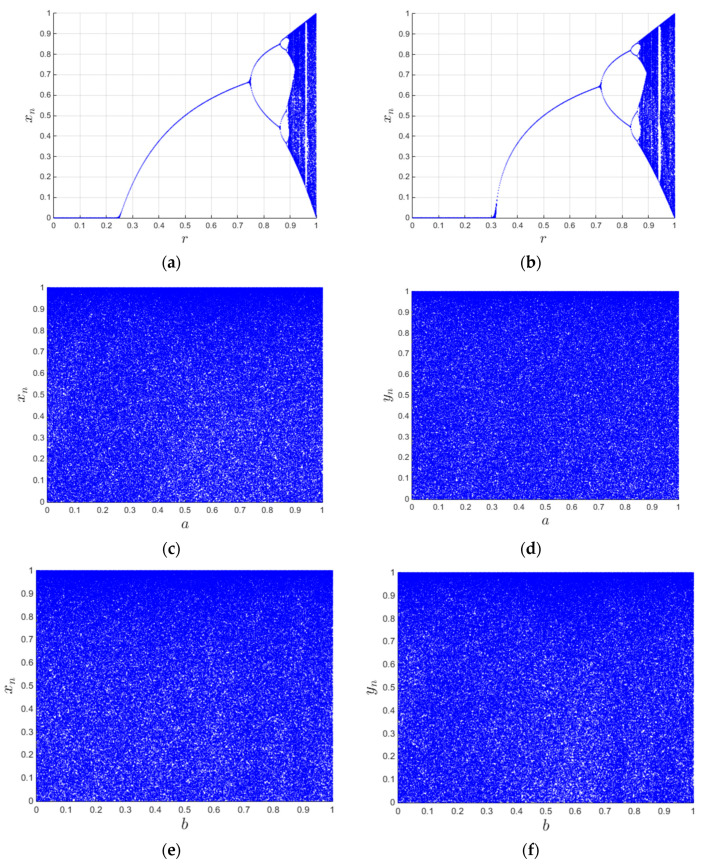
Bifurcation diagrams. (**a**) Bifurcation diagram for the Logistic map (*x*_0_ = 0.5); (**b**) Bifurcation diagram for the Sine map (*x*_0_ = 0.5); (**c**) Bifurcation diagram of *x* for 2D-CACCM (*x*_0_ = 0.2, *y*_0_ = 0.8, *b* = 0.2); (**d**) Bifurcation diagram of *y* for 2D-CACCM (*x*_0_ = 0.2, *y*_0_ = 0.8, *b* = 0.2); (**e**) Bifurcation diagram of *x* for 2D-CACCM (*x*_0_ = 0.2, *y*_0_ = 0.8, *a* = 0.8); (**f**) Bifurcation diagram of *y* for 2D-CACCM (*x*_0_ = 0.2, *y*_0_ = 0.8, *a* = 0.8).

Figure 1a,b describe the bifurcation diagrams of the Logistic map and Sine map separately; their initial values are set as *x*_0_ = 0.5. Figure 1c–f present the bifurcation diagrams of the proposed 2D-CACCM, with the initial values set as *x*_0_ = 0.2 and *y*_0_ = 0.8. Specifically, in Figure 1c,d, the control parameter *b* is fixed at 0.2, and the diagrams depict the variation of state variables *x* and *y* with the control parameter *a*. In Figure 1e,f, the control parameter *a* is fixed at 0.6, and the diagrams illustrate the variation of state variables *x* and *y* with the control parameter *b*. This figure demonstrates that the proposed 2D-CACCM maintains uniform chaotic distribution in the full parameter range, with no obvious periodic windows, which is significantly superior to the classic 1D chaotic maps.

It can be seen from the bifurcation diagrams that the chaotic values are evenly distributed across all intervals of the control parameters. This demonstrates that 2D-CACCM exhibits excellent chaotic characteristics and complex chaotic dynamics over the entire parameter range, which is significantly superior to the classic 1D chaotic maps.

#### 2.2.2. Lyapunov Exponent

**Figure 2:**
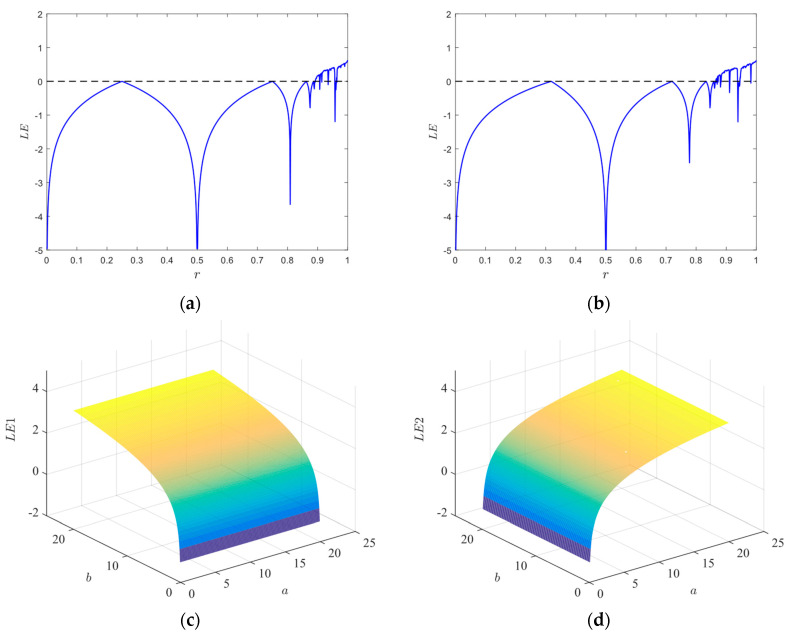
Lyapunov exponents. (**a**) Lyapunov exponents for the Logistic map (*x*_0_ = 0.5); (**b**) Lyapunov exponents for the Sine map (*x*_0_ = 0.5); (**c**) LE of *x* for 2D-CACCM (*x*_0_ = 0.2, *y*_0_ = 0.8); (**d**) LE of *y* for 2D-CACCM (*x*_0_ = 0.2, *y*_0_ = 0.8).

The Lyapunov exponent (LE) is used to quantify the average divergence rate of two trajectories and is a numerical metric for evaluating the complexity of the dynamic system [31]. The LE is defined as follows:

(2) $$\lambda =\underset{n\to \infty }{\lim}\frac{1}{n}\sum_{i=0}^{n-1}ln\left|f^{\prime }(x_{i})\right|,$$

where *f*(*x_i_*) denotes a chaotic system. We calculate the LEs of the proposed 2D-CACCM and conducts a comparative analysis with classic one-dimensional (1D) chaotic maps. As shown in Figure 2a,b, the LEs of the Logistic map and Sine map are positive only within a narrow parameter range, and this positive characteristic is discontinuous, which results in unstable and poor chaotic performance. In contrast, the Lyapunov exponent plots of the 2D-CACCM in Figure 2c,d demonstrate that the proposed system maintains positive LEs over a wide parameter range when *a*, *b* is in (0.1, 20). These results verifies that the 2D-CACCM exhibits excellent and stable chaotic behavior under all tested control parameters.

#### 2.2.3. Spectral Entropy Complexity

Spectral entropy (SE) provides a quantitative measure for assessing the complexity of iterative sequences generated from chaotic dynamics [32].

**Figure 3:**
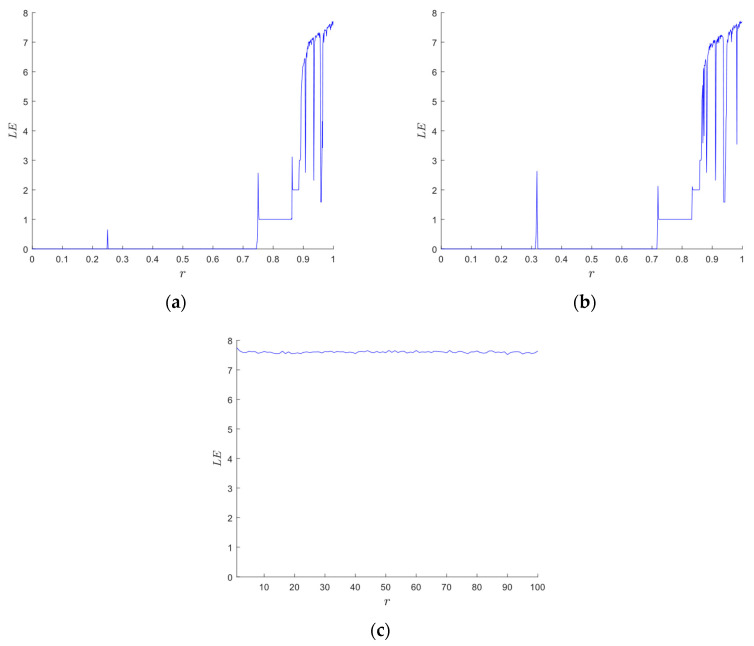
Spectral entropy. (**a**) Spectral entropy for the Logistic map; (**b**) Spectral entropy for the Sine map; (**c**) Spectral entropy for 2D-CACCM.

When SE is positive, the generated sequence is irregular. With the increase in SE, the regularity of the generated sequence decreases, the randomness increases, and the chaotic behavior becomes more complex. The SE of 2D-CACCM is shown in Figure 3, and its SE value is significantly greater than that in the Sine and Logistic maps, indicating that 2D-CACCM has better chaos performance.

#### 2.2.4. Sensitivity

Sensitivity to the initial states means that even small differences in the initial states can result in completely different trajectories in the chaotic map, which is a necessary property for a qualified chaotic system [32]. When we slightly change the initial states of the chaotic map, including the initial values and control parameters, its evolution will quickly deviate from the original trajectory, eventually leading to completely different behavior.

**Figure 4:**
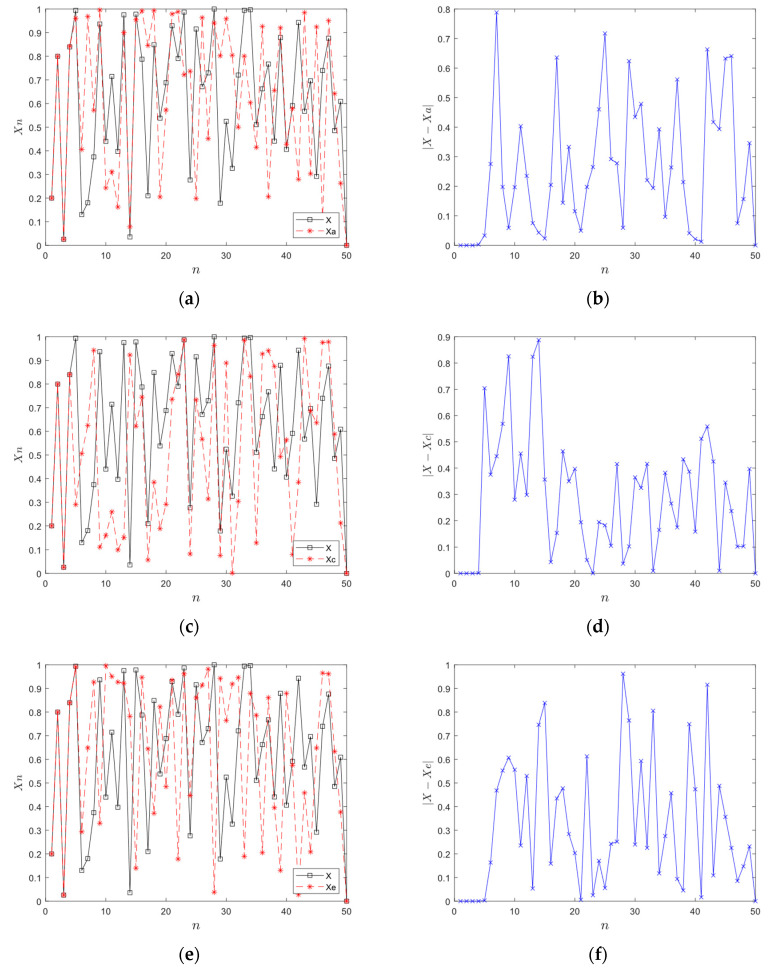
Sensitivity analyses. (**a**) Chaotic trajectories with different *x*_0_; (**b**) Difference of chaos in (**a**); (**c**) Chaotic trajectories with different *x*_1_; (**d**) Difference of chaos in (**c**); (**e**) Chaotic trajectories with different r; (**f**) Difference of chaos in (**e**).

Figure 4a,c show the difference of the output of the chaotic system after 50 iterations with the same control parameters and different initial values, where (*x*_0_, *y*_0_, *a*, *b*) = (0.2, 0.8, 50, 50), (*x*_0*a*_, *x*_1*a*_, *a*_*a*_, *b*_*a*_) = (0.2 + 10^−10^, 0.8, 50, 50) and (*x*_0*b*_, *x*_1*b*_, *a*_*b*_, *b*_*b*_) = (0.2, 0.8 + 10^−10^, 50, 50). Figure 4e describes the difference of the output trajectory with (*x*_0*c*_, *x*_1*c*_, *a*_*c*_, *b*_*c*_) = (0.2, 0.8, 50 + 10^−10^, 50) after 50 iterations. Figure 4b,d,f are the difference values of the chaotic trajectory in (a), (c), and (e), respectively.

It is evident that when the initial state of 2D-CACCM is slightly disturbed, the chaotic trajectory quickly deviates from the original trajectory after a very short overlap, which is negligible in amounts of iteration. It indicates that 2D-CACCM has excellent sensitivity to initial state changes.

#### 2.2.5. The NIST SP800-22 Test

NIST SP800-22 can be used to evaluate the randomness of chaotic sequences in a probabilistic manner [33]. It contains 15 tests, each of which generates a *p*-value. A sequence is deemed to be random if its corresponding *p*-value exceeds 0.01.

A long chaotic sequence of sufficient length is generated by the 2D-CACCM and converted into a binary stream for testing. As can be seen from the test results in Table 1, the bit stream passed all the tests. It can be concluded that the sequences generated by 2D-CACCM are highly randomized.

#### 2.2.6. 0–1 Test

The 0–1 test [34] avoids the need for phase space reconstruction. To determine whether chaos exists in discrete data, one can compute the linear growth rate *K*() of a transformed variable. Given a discrete time series *θ*(*n*), *n* = 1, 2, …, *N* and an arbitrary constant *c* ∈ (0, π), the test statistic *K*(*c*) is obtained as follows:

(3) $$K(c)=corr(\xi ,\Delta )=\frac{\mathrm{cov}(\xi ,\Delta )}{\sqrt{\mathrm{var}(\xi )\mathrm{var}(\Delta )}}\in [-1,1],$$

where

$$\xi =1,\;2,\;\ldots ,\;n,\;\Delta =D_{c}(1),\;D_{c}(2),\;\ldots ,\;D_{c}(n),$$

$$\mathrm{cov}(x,y)=\frac{1}{q}\sum_{i=1}^{q}\left(x(i\right)-\bar{x})(y(i)-\bar{y}),\;\bar{x}=\frac{1}{q}\sum_{i=1}^{q}x(i),\;\mathrm{var}(x)=\mathrm{cov}(x,x),$$

$$D_{c}(n)=\underset{n\to \infty }{\lim}\frac{1}{N}\sum_{i=1}^{N}({\left(p_{c}(i+n)-p_{c}(i)\right)}^{2}+{\left(q_{c}(i+n)-q_{c}(i)\right)}^{2})-{\left(E(\theta )\right)}^{2}\frac{1-\cos(nc)}{1-\cos(c)},$$

and

$$p_{c}(n)=\sum_{i=1}^{n}\theta (i)\cos(ic),\;q_{c}(n)=\sum_{i=1}^{n}\theta (i)\sin(ic),\;E(\theta )=\underset{N\to \infty }{\lim}\frac{1}{N}\sum_{i=1}^{N}\theta (i).$$

**Figure 5:**
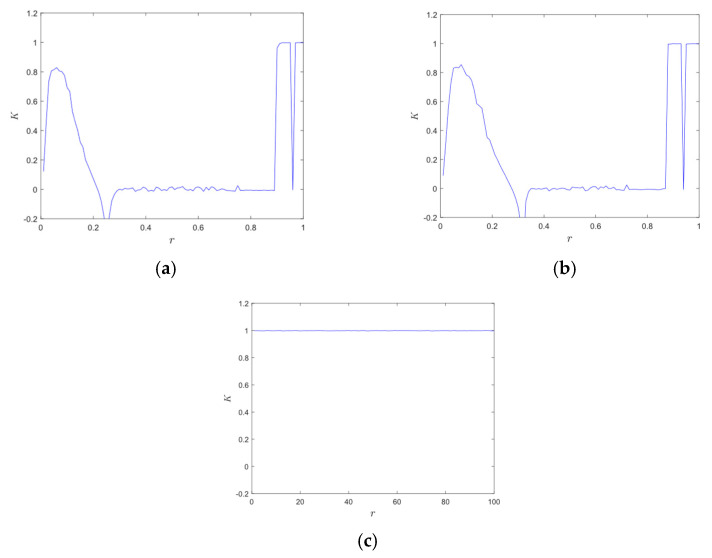
0–1 test results. (**a**) 0–1 test for Logistic map; (**b**) 0–1 test for Sine map; (**c**) 0–1 test for 2D-CACCM.

The closer the value of *K* is to 1, the more chaotic the sequence is. With *N* = 100 and *c* ∈ (π/5, 4π/5), the corresponding 0–1 test results are shown in Figure 5. The *K* value of the Logistic map and Sine map fluctuate and are close to 1 in a small range. The 0–1 test results of 2D-CACCM converge to 1 for all parameters. Therefore, 2D-CACCM has better chaos characteristics.

### 2.3. Image 3D Histogram Model

Traditional image encryption algorithms typically adopt the scrambling–diffusion structure, which requires two distinct operational phases. In recent years, simultaneous scrambling–diffusion has emerged as an active research direction, enabling the integrated permutation of pixel positions and the alteration of pixel values in a single phase.

**Figure 6:**
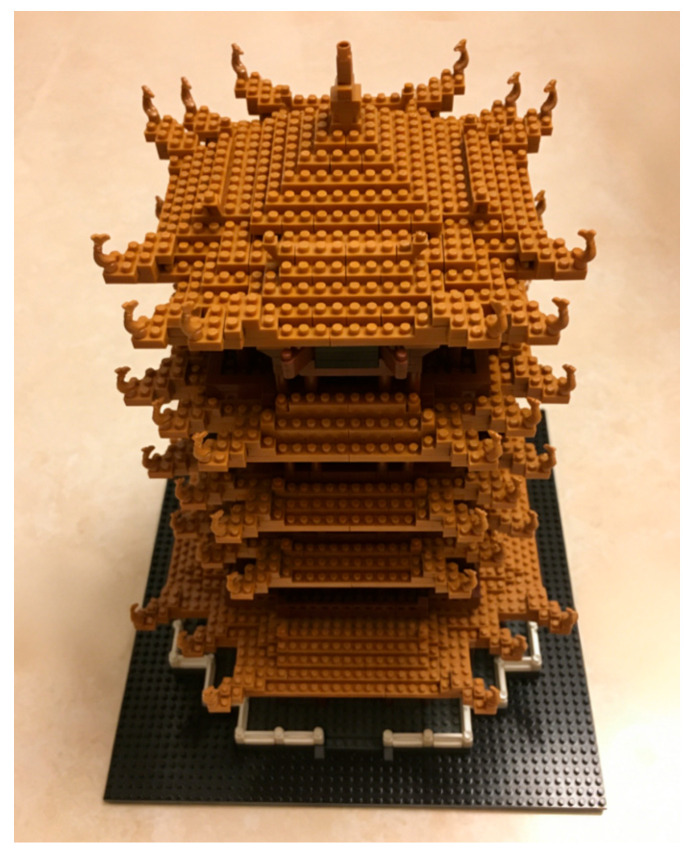
The particle tower model.

Studies have shown that the 3D histogram of a digital image and the particle tower model share structural similarities in their 3D architectures, both exhibiting a 3D distribution consisting of spatial coordinates and grayscale values (or particle-stacking properties). Figure 6 shows a particle tower model. Through our design, this 3D structure can be utilized to achieve simultaneous scrambling–diffusion for digital images.

**Figure 7:**
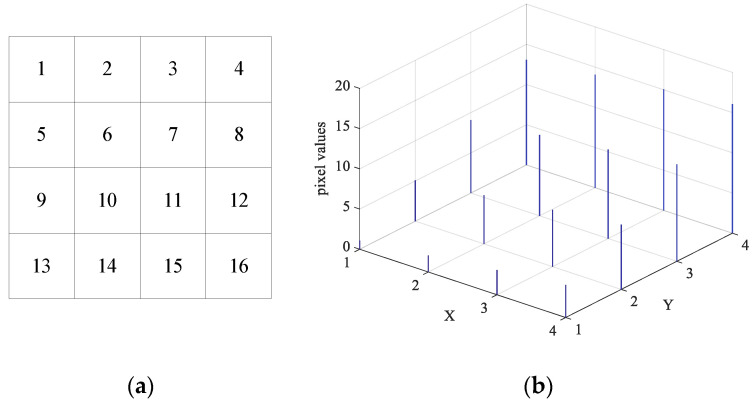
Expanding process of 4 × 4 original image. (**a**) 4 × 4 original image (Numbers 1–16 represent pixels at the corresponding positions); (**b**) 3D histogram presentation of original image.

**Figure 8:**
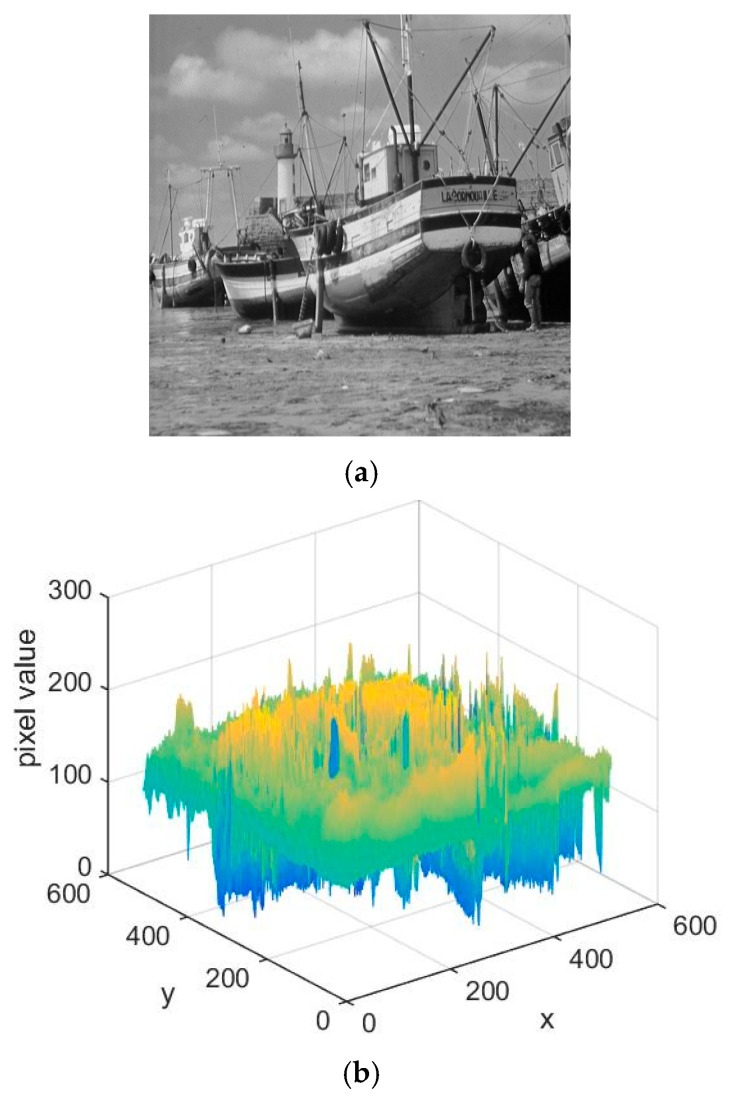
The ‘Boat’ and its 3D histogram presentation. (**a**) The plaintext ‘Boat’; (**b**) 3D histogram presentation of Boat.

A 3D histogram-based image encryption algorithm is defined as an algorithm that extends the dimensionality of a digital image, modifies the pixel positions and values in the image matrix from the perspective of 3D histograms, and thereby completes the scrambling and diffusion processes. A 2D digital image is converted into a 3D representation and is subsequently observed and encrypted from this three-dimensional perspective: each point on the plane with pixel value *p* can be seen as a stack of *p* particles, and the entire image can be seen as consisting of 256 grayscale planes. Figure 7a shows the original 4 × 4 2D grayscale image, where each grid represents a pixel with its corresponding grayscale value. Figure 7b presents the 3D histogram representation of the 4 × 4 image, where the X and Y axes correspond to the spatial coordinates of pixels in the original 2D image, and the Z axis corresponds to the grayscale value of the pixel at the corresponding coordinate. Figure 8 shows the ‘Boat’ image and its 3D format.

The specific steps for extending the plain image to 3D space and merge to 2D are described as follows:

Step 1: Acquiring the image size parameters

Read the original image *I* and acquire its size parameters using

(4) [m,n]=size(I).

Step 2: Generating a 3D zero matrix

Create a 3D zero matrix $I^{\prime }$ with the size of *m* × *n* × 256.

(5) $$I^{\prime }=zeros(m,n,256).$$

Step 3: Traversing and filling the 3D space

Traverse the original image *I*, and assign the grayscale value *I*(*x*, *y*) to *I′*(*x*, *y*, *I*(*x*, *y*) + 1).

(6) $$I^{\prime }(x,y,I(x,y)+1)=1,x=1,2,\ldots ,m;y=1,2,\ldots ,n.$$

Given that MATLAB’s matrix indices start at 1, the grayscale value is incremented by 1 to align with this convention. This adjustment ensures that index values correspond accurately to MATLAB’s indexing scheme when manipulating matrices or arrays in its environment.

Step 4: Permuting pixel positions and diffuse grayscale values

Perform 3D simultaneous permutation and diffusion using

(7) $$I^{\prime \prime }(f(x),g(y),h(s))=I^{\prime }(x,y,s),x=1,2,\ldots ,m;y=1,2,\ldots ,n,$$

where *f* (·) and *g*(·) denote spatial permutation functions acting on the coordinate system, and *h*(·) denotes the intensity diffusion function operating on grayscale values. The proposed algorithm uses s=find(A(x,y,:)) to acquire the *z* index and reduce computational complexity.

Step 5: Merging the 3D matrix and recover the original image.

Since the 3D matrix consists of zeros and the original grayscale values in the *z* direction, the original pixel value can be calculated by summing the values along the *z*-axis.

(8) $$I^{\prime \prime \prime }=sum(I^{\prime \prime }(:,:,s)\times (s-1)),s=1,2,\ldots ,256.$$

To maintain consistency with Equation (6) and successfully recover the plaintext image, a subtraction of 1 is applied to *s*. Algorithm 1 describes the extension and merging process based on the 3D model, where notations are represented by ‘%’ in MATLAB language.
**Algorithm 1:** Extending and merging based on the 3D histogram model**Input:** the plain image *I* and the chaos sequences *f* (·), *g* (·), and *h* (·)
**Output:** the permuted matrix *E*  1: % Extending
  2: [*m*, *n*] ← size(*I*)
  3:$\;I^{\prime }$ ← zeros(*m*, *n*, 256)
  4: **for**
*i* ← 1 to *m*
**do**
  5:    **for**
*j* ← 1 to *n*
**do**
  6:    
$\;I^{\prime }$(*i*, *j*, *I*(*i*, *j*) + 1) ← 1
  7:    **end**
  8: **end**
  9: **for**
*i* ← 1 to *m*
**do**
10:    **for**
*j* ← 1 to *n*
**do**
11:      *s* ← find(*I*’(*i*, *j*,:))
12:     
$I^{\prime \prime }(f(i),g(j),h(s))\leftarrow I^{\prime }(i,j,s)$
13:    **end**
14: **end**
15: % Merging
16:
$\;[m,\;n,\;\sim ]\;\leftarrow \;\mathrm{size}(I^{\prime }$)
17: **for** k ← 1 to *256*
**do**
18:    *E* ← *sum*(*I′*(:, :, k) × (k − 1))
19: **end**

## 3. Proposed Image Encryption Algorithm

A communication scenario for encryption and decryption is first defined: the sender and receiver are denoted as Alice and Bob, respectively. The following sections describe the core techniques of the proposed algorithm.

### 3.1. Alice’s Key Generation Process

The Secure Hash Algorithm 256-bit (SHA-256) is a standardized cryptographic hash function issued by the National Institute of Standards and Technology in the Federal Information Processing Standards Publication 180-4, and is the core member of the SHA-2 family. It accepts an input message of arbitrary length, processes it through an iterative Merkle–Damgård compression structure, and outputs a fixed-length 256-bit irreversible message digest [35]. This paper combines the generated hash value with user-defined external keys to jointly calculate the initial values and control parameters of the 2D-CACCM chaotic system, so that the final encryption sequence is not only strongly related to the plaintext content, but also can be flexibly adjusted via external keys. Meanwhile, this design greatly expands the key space of the algorithm and further guarantees encryption security.

The proposed algorithm first applies the SHA-256 hash algorithm to compute the hash value *K_h_* of the plaintext image. It then generates the control parameters and initial values of 2D-CACCM by combining *K_h_* with the external keys. The detailed steps for key generating are as follows.

Step 1: Blocking the hash value

Split the 256-bit hash value *K_h_* into 32 equal parts, each 8 bits in length. Let *K_h_* = {*k*_1_, *k*_2_, …, *k*_32_}, with each *k_i_* being an 8-bit binary value.

Step 2: Calculating intermediate parameters

The 32 segments are XORed with each other, and four intermediate parameters are derived by following equations:

(9) $$\left\{\begin{array}{l} h_{1}=bin2dec(\frac{k_{1}+k_{5}+k_{9}+k_{13}+k_{17}+k_{21}+k_{25}+k_{29}}{8})\\ h_{2}=bin2dec(\frac{k_{2}+k_{6}+k_{10}+k_{14}+k_{18}+k_{22}+k_{26}+k_{30}}{8})\\ h_{3}=bin2dec(\frac{k_{3}+k_{7}+k_{11}+k_{15}+k_{19}+k_{23}+k_{27}+k_{31}}{8})\\ h_{4}=bin2dec(\frac{k_{4}+k_{8}+k_{12}+k_{16}+k_{20}+k_{24}+k_{28}+k_{32}}{8}) \end{array}\right.,$$

where ⨁ represents the XOR operation.

Step 3: Generating encryption keys

The control parameters *a*, *b* and the initial values *x*_0_, *y*_0_ of the 2D-CACCM are computed by

(10) $$\left\{\begin{array}{l} a=(e_{1}+mod(h_{1},100))/2\\ b=(e_{2}+mod(h_{1},100))/2\\ x_{0}=e_{3}/2+h_{2}/512+h_{3}/512\\ y_{0}=e_{4}/2+h_{4}/256 \end{array}\right.,$$

where $e_{1},\;e_{2}\in (0,\;100)$ and $e_{3},\;e_{4}\in (0,\;1)$ are the user-specific external keys. The generated $a,\;b,\;{\;x}_{0}\;{,\;y}_{0},$ are the keys in our algorithm that need to be sent to the receiver along with the encrypted image during data transmission.

### 3.2. Alice’s Encryption Process

**Figure 9:**
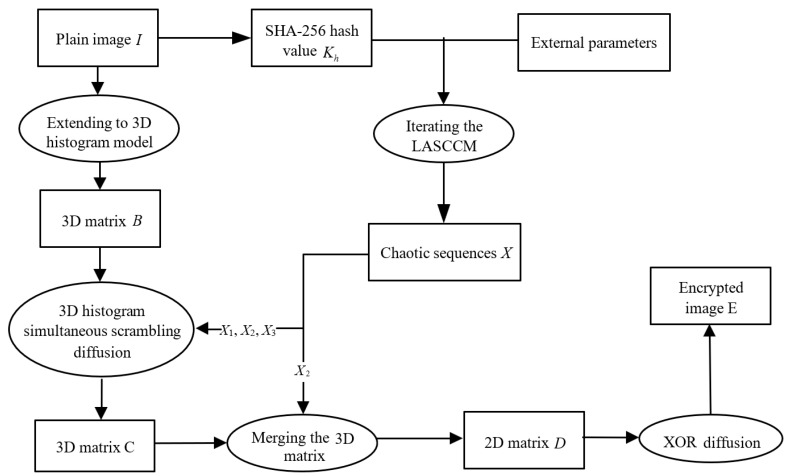
Flowchart of the encryption process.

The algorithm adopts a simultaneous scrambling–diffusion method based on the 3D histogram model; the flowchart is shown in Figure 9. Firstly, the 3D matrix is obtained by extending the plaintext image in accordance with Algorithm 1. Secondly, the chaotic sequences are used for simultaneous scrambling–diffusion. Thirdly, the 3D matrix is merged to obtain the 2D image. Finally, the 2D XOR diffusion is performed to achieve further diffusion to obtain a final encrypted image. Algorithm 2 shows the encryption process, and the detailed steps are as follows.

**Algorithm 2:** The encryption process**Input:** the initial image *I* and the external keys **Output:** the encrypted image *E* 1: % Calculate the initial values and control parameters of the chaotic sequence 2: *K_h_* ← SHA256(*I*) 3: {*k*_1_, *k*_2_, …, *k*_32_} ← *K_h_* 4: for *i* ← 1 to 4 do
5: *h_i_*$\;\leftarrow \;bin2dec(k_{i}⨁k_{4+i}⨁k_{8+i}⨁k_{12+i}⨁k_{16+i}⨁k_{20+i}⨁k_{24+i}⨁k_{28+i})$
6: end
7: *a* ← ($e_{1}$ + *mod*(*h*_1_,100))/2
8:
b ← ($e_{2}$ + *mod*(*h*_1_,100))/2 9: *x*_0_ ← $e_{3}/2$ + *h*_2_/512 + *h*_3_/512
10: *y*_0_ ← $e_{4}/2$ + *h*_4_/256 11: % Generate the chaotic sequences *X*_1_, *X*_2_ with the length of *mn*
12: *X*_1_, *X*_2_ ← 2D-CACCM($a,\;{b,\;x}_{0},\;y_{0},\;mn$)
13: *X*_3_ ← [*X*_1_(1:128), *X*_2_(*mn* − 127, *mn*)]
14: [~,*Y*_1_] ← *sort*(*X*_1_(1:*m*))
15: [~,*Y*_2_] ← *sort*(*X*_2_(*mn* − n + 1:*mn*))
16: [~,*Y*_3_] ← *sort*(*X*_3_)
17: % Extending to 3D space
18: *B* ← zeros(*m*, *n*, 256)
19: **for**
*i* ← 1 to *m*
**do**
20:    **for**
*j* ← 1 to *n*
**do**
21:     *B*(*i*, *j*, *I*(*i*, *j*) + 1) ← 1
22:    **end**
23: **end**
24: % 3D simultaneous scrambling–diffusion
25: **for**
*j* ← 1 to *n*
**do**
26:    **for** I ← 1 to *m*
**do**
27:      *s* ← find(*B*(*i*, *j*,:))
28:      $C(i,j,s)\leftarrow B(Y_{1}(i),Y_{2}(j),Y_{3}(s))$
29:    **end**
30: **end**
31: % Merging to 2D image
32: for *k* ← 1 to 256 do
33:    *D* ← sum(*C*(:, :, *k*) × (*k* − 1))
34: end
35: %2D XOR diffusion
36: *Z* ← *mod*(floor(*X*_2_ × 10^15^), 256)
37: **for**
*i* ← 1 to *mn*
**do**
38:    E(i,j)←bitxor(D(i,j),Z(j+(i−1)×m)),i=1,2,…,m,j=1,2,…,n.
39: **end**

Step 1: Generating encryption keys

The SHA-256 algorithm is applied to the plaintext image *I*_*m*×*n*_ to produce a 256-bit hash value *K_h_*. Following the procedure in Section 3.1, the externally supplied keys are random real numbers that serve as the basis for calculating the control parameters and initial values of the 2D-CACCM.

Step 2: Generating chaotic sequences

Alice iterates the 2D-CACCM for 1000 + *m* × *n* iterations. The first 1000 non-chaotic values are discarded. Chaotic sequences *X*_1_ and *X*_2_, each with a length of *m* × *n*, are thus obtained.

Step 3: Separating and sorting chaotic sequences

The chaotic sequence *X*_3_ is derived from *X*_1_, *X*_2_ as follows:

(11) $$X_{3}=\left[X_{1}(1:128),X_{2}(mn-127:mn)\right].$$

Step 4: Extending plaintext image based on the 3D histogram model

The matrix *I* is extended using the 3D histogram model described in Section 2.3, yielding the extended 3D matrix *B*. For the sake of subsequent processing, Equation (6) is revised as follows:

(12) B(x,y,I(x,y)+1)=1,x=1,2,…,m;y=1,2,…,n.

Step 5: Performing 3D histogram simultaneous scrambling–diffusion

3D histogram simultaneous scrambling–diffusion is performed by Equation (14), and the resulting matrix is denoted as *C*.

(13) $$\left\{\begin{array}{l} (\sim ,Y_{1})=sort(X_{1}(1:m)),\\ (\sim ,Y_{2})=sort(X_{2}(mn-n+1:mn)),\\ (\sim ,Y_{3})=sort(X_{3}), \end{array}\right.$$

(14) $$C(x,y,s)=B(Y_{1}(x),Y_{2}(y),Y_{3}(s)),x=1,2,\ldots ,m;y=1,2,\ldots ,n;s=1,2,\ldots ,256,$$

where *Y*_1_, *Y*_2_ are used to scramble 3D matrix *B* and *Y*_3_ is used to diffuse the 3D matrix *B*.

Step 6: Merging to 2D image

The matrix *D* is merged in accordance with Step 4 in Section 2.3. Due to the revision of Equation (6), Equation (8) is also adjusted to

(15) D=sum(C(:,:,s)×(s−1)),s=1,2,…,256.

Step 7: Performing 2D XOR diffusion

2D XOR diffusion operation is performed on the matrix *D* after simultaneous scrambling–diffusion using Equation (17).

(16) $$Z=mod(floor(X_{2}\times 10^{15}),256)$$

(17) E(i,j)=bitxor(D(i,j),Z(j+(i−1)×m)),i=1,2,…,m,j=1,2,…,n.

The resulting matrix *E* with the size of *m* × *n* is the final encrypted image.

### 3.3. Bob’s Decryption Process

**Figure 10:**
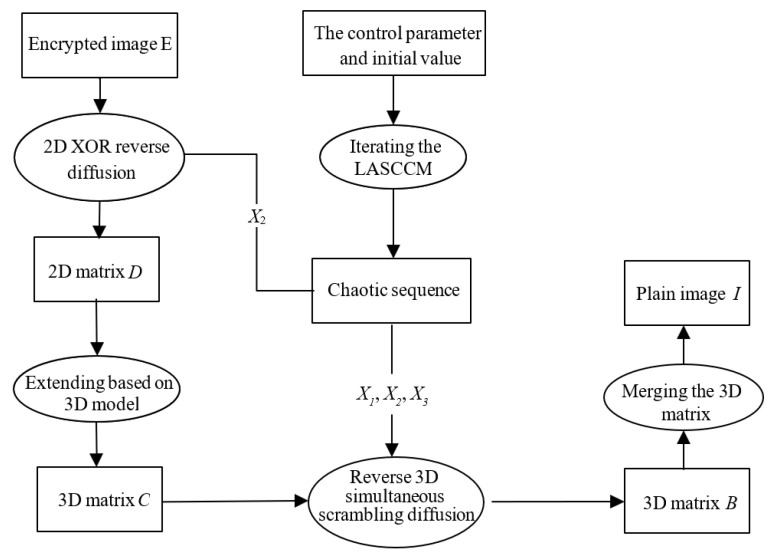
Flowchart of the decryption process.

The decryption process is the strictly symmetric inverse operation of the encryption process, and the correctness of decryption is guaranteed by the reversibility of all operations in the encryption framework. The receiver Bob needs to use the exact same control parameters *a*, *b* and initial values *x*_0_, *y*_0_ of the 2D-CACCM to regenerate the completely consistent chaotic sequences, and then execute the inverse operations in the reverse order of the encryption process. Specifically, the reverse 2D XOR diffusion corresponds to the 2D XOR diffusion step in the encryption process, the reverse 3D simultaneous scrambling–diffusion corresponds to the 3D histogram simultaneous scrambling–diffusion step, and the 3D matrix merging corresponds to the 2D-to-3D extension step. Figure 10 illustrates the overall flow of the decryption process, and Algorithm 3 lists each step in detail. The detailed execution steps of the full decryption process are described below.

**Algorithm 3:** The decryption process**Input:** the encrypted image *E*, the initial values and control parameters of chaotic map **Output:** the decrypted image *I*
 1: % Generate the chaotic sequences *X*_1_, *X*_2_ with the length of mn
 2: *X*_1_, *X*_2_ ← 2D-CACCM($a,b,x_{0},y_{0},mn$)
 3: *X*_3_ ← [X1(1:128), X2(mn − 127, mn)]
 4: [~, *Y*_1_] ← sort(*X*_1_ (1:m))
 5: [~, *Y*_2_] ← sort(*X*_2_ (mn − *n* + 1:mn))
 6: [~, *Y*_3_] ← sort(*X*_3_)
 7: % Reverse 2d XOR diffusion
 8: Z ← *mod*(floor(*X*_2_ × 10^15^), 256)
9: **for**
*i* ← 1 to *m*
**do**
10:        **for**
*j* ← 1 to *n*
*do*
11:    D(i,j)←bitxor(E(i,j),Z(j+(i−1)×m))
    12:  end 13: **end** 14: % Extending to 3D space
15: *C* ← zeros(*m*, *n*, 256)
16: **for**
*i* ← 1 to *m*
**do**
17:    **for**
*j* ← 1 to *n*
**do**
18:      *C*(*i*, *j*, *D*(*i*, *j*) + 1) ← 1
19:    **end**
20: **end**
21: % Reverse 3D simultaneous scrambling–diffusion
22: **for** j ← 1 to *n*
**do**
23:    **for** i ← 1 to *m*
**do**
24:      *Y*_3_(*s*) ← find(*C*(*i*, *j*,:))
25:      $B(i,j,s)\leftarrow C(Y_{1}(i),Y_{2}(j),Y_{3}(s))$
26:    **end**
27: **end**
28: % Merging to plain image
29: **for**
*i* ← 1 to 256 **do**
30:      *I* ← sum(*B*(:, :, *i*) × (i − 1)) 31: **end**

Step 1: Generating chaotic sequences

Bob iterates the 2D-CACCM for 1000 + *mn* iterations using the keys *a*, *b*, *x*_0_, *y*_0_, and discards the first 1000 values to obtain chaotic sequences *X*_1_, *X*_2_ with a length of *m* × *n*.

Step 2: Separating chaotic sequences and deriving index sequences

Three chaotic sequences—*X*_1_, *X*_2_ and *X*_3_—are obtained according to Equation (11). Ascending index sequences *Y*_1_, *Y*_2,_ and *Y*_3_ are obtained from Equation (13), and *Z*_1_ is obtained from Equation (16).

Step 3: Performing reverse 2D XOR diffusion

Reverse 2D XOR diffusion is performed by Equation (18), yielding the matrix D.

(18) D(i,j)=bitxor(E(i,j),Z(j+(i−1)×m)),i=1,2,…,m,j=1,2,…,n.

Step 4: Extending encrypted image based on 3D histogram model

The matrix *D* is extended by Equation (19), yielding the 3D matrix *C*.

(19) C(x,y,D(x,y)+1)=1,x=1,2,…,m;y=1,2,…,n.

Step 5: Performing reverse 3D simultaneous scrambling–diffusion

Equation (20) is used to inversely permutate the 3D matrix *C*, and the result is denoted as *B*.

(20) $$B(Y_{1}(x),Y_{2}(y),Y_{3}(s))=C(x,y,s),x=1,2,\ldots ,m;y=1,2,\ldots ,n;s=1,2,\ldots ,256,$$

Step 6: Merging to recover original plaintext image

The matrix *B* is merged by Equation (21) and the resulting matrix is denoted as *I*—the recovered original plaintext image.

(21) I=sum(B(:,:,s)×(s−1)),s=1,2,…,256.

## 4. Simulation Experiments

**Figure 11:**
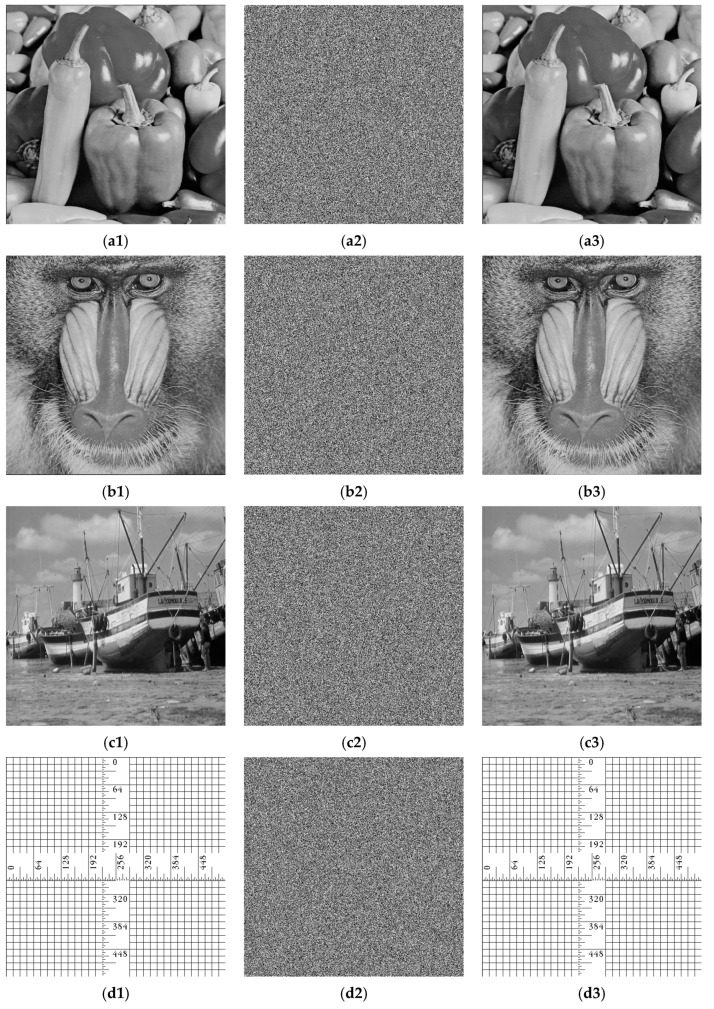
Simulation results: (**a1**–**d1**) Plaintext images; (**a2**–**d2**) Ciphertext images; (**a3**–**d3**) Decrypted images.

To validate the effectiveness of the proposed encryption scheme, a series of simulations were conducted using MATLAB R2018a. The experimental environment consisted of a PC equipped with a 2.9 GHz Intel Core i5-9400F processor, 8.00 GB of RAM, and the 64-bit version of Windows 10. Test images were sourced from the USC-SIPI image archive “http://sipi.usc.edu/database” (accessed on 25 March 2026) [36] as well as other commonly employed standard test images. Four grayscale images, each with a size of 512 × 512, were used in the experiments. Taking the Peppers image as an example, its 256-bit hash value was computed as *K_h_* = 5bbf26243660100464e2bc8f6f487367eb238ce0c30 9cc2fa9e587c693276c9c. The external keys were randomly selected as *e*_1_ = 43.96777, *e*_2_ = 43.96778, *e*_3_ = 0.0022, and *e*_4_ = 0.6323. From these, the resulting control parameters and initial values were determined: *a* = 29.9839, *b* = 39.9652, *x*_0_ = 0.4679, and *y*_0_ = 0.6794. Figure 11 presents the original plaintext images, their corresponding ciphertext images, and the decrypted results. As can be seen, no meaningful information can be extracted from the encrypted images, confirming the efficacy of the proposed algorithm.

## 5. Algorithm Analyses

The rapid development of chaotic image encryption has necessitated comprehensive evaluation to assess security performance. Recent reviews in the literature systematically categorize critical evaluation parameters into four dimensions: statistical robustness (histogram uniformity, correlation dissipation, information entropy), differential attack resilience (NPCR/UACI), computational security (key space, sensitivity), and implementation efficiency. To fully prove the stability of the algorithm, we have carried out experiments and analyzed the algorithm considering these aspects.

### 5.1. Sensitivity Analyses

#### 5.1.1. Key Space Analysis

The key space is the sum of all possible values of unknown parameters in the algorithm, and its size determines the ability of the encryption algorithm to resist exhaustion attacks. If the key space is too small, the attacker may infer the initial key by violent enumeration, and thus crack the encryption algorithm. A key space exceeding 2^100^ is generally considered large enough to resist exhaustive search attacks [37]. The key space of our algorithm is discussed below in terms of two aspects.

(1) The view of the hash value and external keys

The hash value *K_h_* is a 256-bit binary sequence, and the external keys are $e_{1}-e_{4}$. As is mentioned in Section 3.1, $e_{1}{,e}_{2}\in (0,\;100)$ and $e_{3}{,e}_{4}\in (0,\;1)$ are set in the experiment. Assuming a computational precision of 10^−14^, the total key space of our algorithm is approximately 2^256^ × 100 × 10^14^ × 100 × 10^14^ × 10^14×2^ ≈ 2^455^.

(2) The view of the chaotic system

The control parameters are *a*, *b* ∈ (0, 100) and the initial values are *x*_0_, *y*_0_ ∈ (0, 1). Assuming a computational precision of 10^−14^, the total key space of our algorithm is approximately 100 × 10^14^ × 100 × 10^14^ × 10^14×2^ ≈ 2^199^.

Taking both of the above considerations into account, the minimum key space of our algorithm reaches 2^199^, which is far greater than the generally accepted threshold of 2^100^ required for cryptographic security. Table 2 compares the key space of the proposed scheme with those of several other algorithms. Although our algorithm does not outperform all existing methods in this particular metric, it is nevertheless sufficiently large to withstand brute-force attacks.

#### 5.1.2. Key Sensitivity Analysis

**Figure 12:**
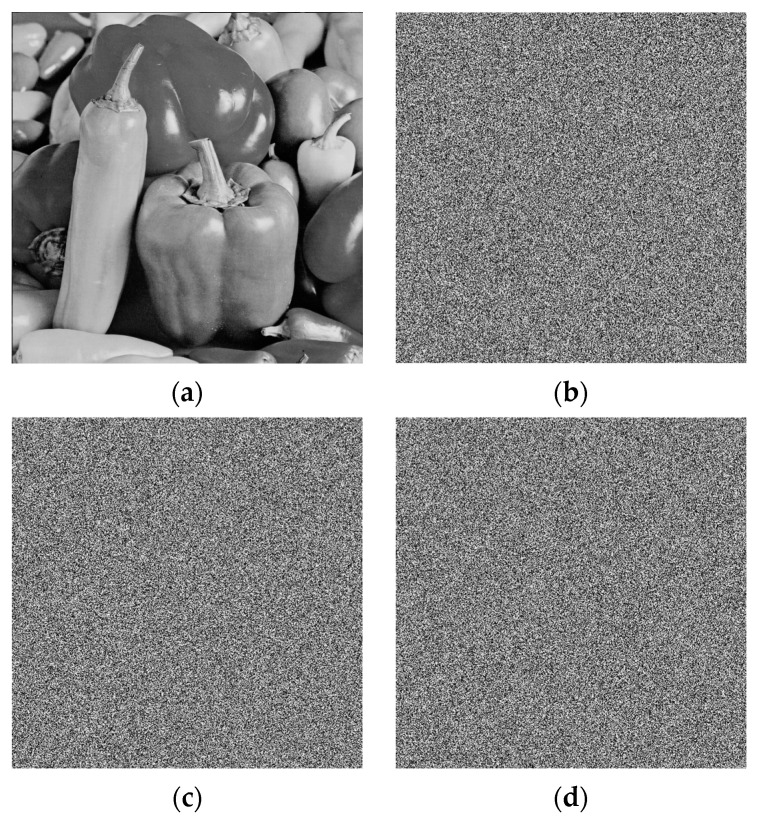
Decrypted images with the correct and the wrong keys. (**a**) Decrypted image with the correct *key*_0_ = {*a*, *b*, *x*_0_, *y*_0_}; (**b**) Decrypted image with the wrong *key*_1_ = {*a* + 10^−14^, *b*, *x*_0_, *y*_0_}; (**c**) Decrypted image with the wrong *key*_2_ = {*a*, *b*, *x*_0_ + 10^−14^, *y*_0_}; (**d**) Decrypted image with the wrong *key*_3_ = {*a*, *b*, *x*_0_, *y*_0_ + 10^−14^}.

A good algorithm must have high key sensitivity to defend against known plaintext attacks. In the analysis of key sensitivity, a pair of security keys containing small differences are used to encrypt the same original image to generate two completely different cryptographic images, and thus the difference between the two cryptographic images is further analyzed [39]. To verify the key sensitivity of the proposed encryption scheme visually, we test the decrypted results using the correct key and slightly perturbed keys, supposing that the key utilized in the proposed algorithm is *key*_0_ = {*a*, *b*, *x*_0_, *y*_0_}. Then, 10^−14^ increases are made to *r*, *x*_0_, and *x*_1_, respectively. The three new key sets are given as follows: *key*_1_ = {*a* + 10^−14^, *b*, *x*_0_, *y*_0_}, *key*_2_ = {*a*, *b*, *x*_0_ + 10^−14^, *y*_0_}, and *key*_3_ = {*a*, *b*, *x*_0_, *y*_0_ + 10^−14^}. The encrypted image is then decrypted separately using the three altered key sets. As illustrated in Figure 12, all decrypted images with perturbed keys are completely noise-like and cannot recover any valid plaintext information, which directly verifies the ultra-high key sensitivity of the proposed encryption algorithm. Table 3 provides a numerical comparison among them. Even a tiny change in the secret key completely destroys the decrypted image and cannot recover valid plaintext information, which fully demonstrates the high key sensitivity of the proposed algorithm.

#### 5.1.3. Differential Attack Analysis

The resistance of an encryption algorithm against differential cryptanalysis is usually quantified using the Number of Pixels Change Rate (NPCR) and Uniform Average Change Intensity (UACI) [42]. Their mathematical definitions are provided below.

(22) $$D(i,j)=\left\{\begin{array}{l} 0,c_{1}(i,j)=c_{2}(i,j)\\ 1,c_{1}(i,j)\neq c_{2}(i,j) \end{array}\right.,$$

(23) $$NPCR=\frac{1}{m\times n}\times \sum_{i=1}^{m}\sum_{j=1}^{n}D(i,j)\times 100\%,$$

(24) $$UACI=\frac{1}{255\times m\times n}\times \sum_{i=1}^{m}\sum_{j=1}^{n}\left|c_{1}(i,j)-c_{2}(i,j)\right|\times 100\%,$$

where *c*_1_(*i*, *j*) denotes a pixel from the ciphertext image obtained from the original plaintext, while *c*_2_(*i*, *j*) corresponds to the pixel at the same position in the ciphertext image generated after a single-pixel modification in the plaintext. The ideal theoretical values for NPCR and UACI are 99.6094% and 33.4635%, respectively [41].

In this test, randomly chosen pixels in the plain image are altered at arbitrary positions. Because different image sizes may affect the test outcomes [43], we conducted experiments on multiple images of various sizes, with the results listed in Table 4. The obtained NPCR and UACI figures approximate the theoretical ideals well. Therefore, our algorithm meets the NPCR and UACI requirements and is capable of resisting differential attacks. As can be observed in Table 5, the performance of the proposed scheme is closer to the theoretical values than that of other algorithms.

### 5.2. Statistical Analyses

#### 5.2.1. Histogram Analysis

**Figure 13:**
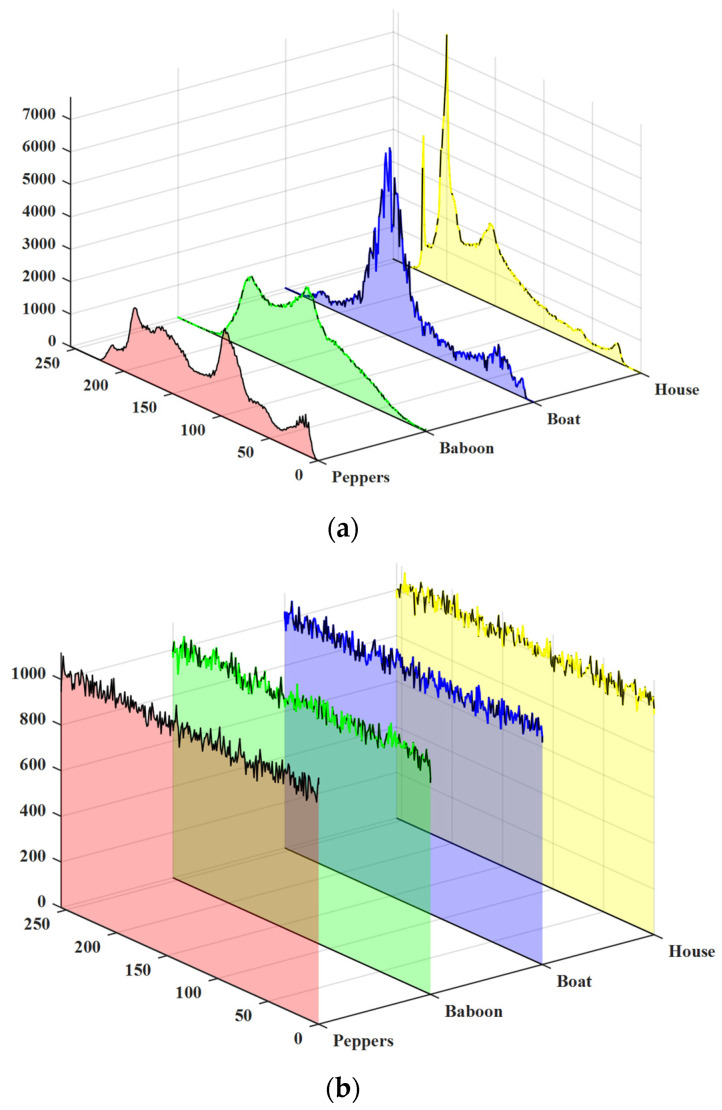
Histograms of plaintext and their corresponding ciphertext. (**a**) Histograms of original images; (**b**) Histograms of ciphertext.

The histogram is the statistic of the distribution frequency of image gray values, which is the most basic statistical feature of an image. If the histogram distribution has obvious statistical properties, an attacker may be able to infer plaintext image information from it. The ciphertext histogram of a good encryption algorithm should be uniform and undifferentiated. The Peppers, Baboon, Boat, and House images are selected, and the histograms of plain images and their corresponding cipher images are shown in Figure 13. Obviously, the histograms of the encrypted images are more uniform and dispersed than those of the ordinary images, which indicates that the algorithm has good anti-statistical attack ability.

#### 5.2.2. Chi-Squared Test

To avoid visual errors and quantitatively analyze these values more accurately, we introduce a Chi-square test to give a statistical representation of pixel uniformity between gray values.

The Chi-square is defined by

(25) $$\chi^{2}=\sum_{L=0}^{255}\frac{{\left(o_{L}-e_{L}\right)}^{2}}{e_{L}},L=0,1,\ldots ,255,$$

where *o_L_* and *e_L_* are the observed and the expected numbers of the *L*-th gray level, respectively.

The Chi-square test results for both the plaintext and ciphertext images are presented in Table 5. As shown in Table 6, the χ^2^ values of all encrypted images fall below the critical values at the 1% and 5% significance levels [46].

#### 5.2.3. Correlation Analysis

A characteristic feature of digital images is the strong correlation typically observed between neighboring pixels, which attackers may exploit to extract meaningful information from textural patterns. An effective image encryption algorithm should therefore be capable of breaking such correlations. To evaluate this capability, we randomly select 1000 adjacent pixel pairs from both the plaintext and its corresponding ciphertext, considering horizontal, vertical, and diagonal directions. The correlation coefficient is then computed using the following formula:

(26) $$E(x)=\frac{1}{m}\sum_{i=1}^{m}x_{i},$$

(27) $$D(x)=\frac{1}{m}\sum_{i=1}^{m}{\left(x_{i}-E(x)\right)}^{2},$$

(28) $$r_{x,y}=\frac{E((x-E(x))(y-E(y)))}{\sqrt{D(x)D(y)}},$$

where variables *x* and *y* correspond to the grayscale values of neighboring pixels in either the horizontal, vertical, or diagonal orientation. The quantity mm indicates the size of the selected pixel sample, *D*(*x*) denotes the variance of *x*, and *E*(*x*) is the expectation of *x*.

**Figure 14:**
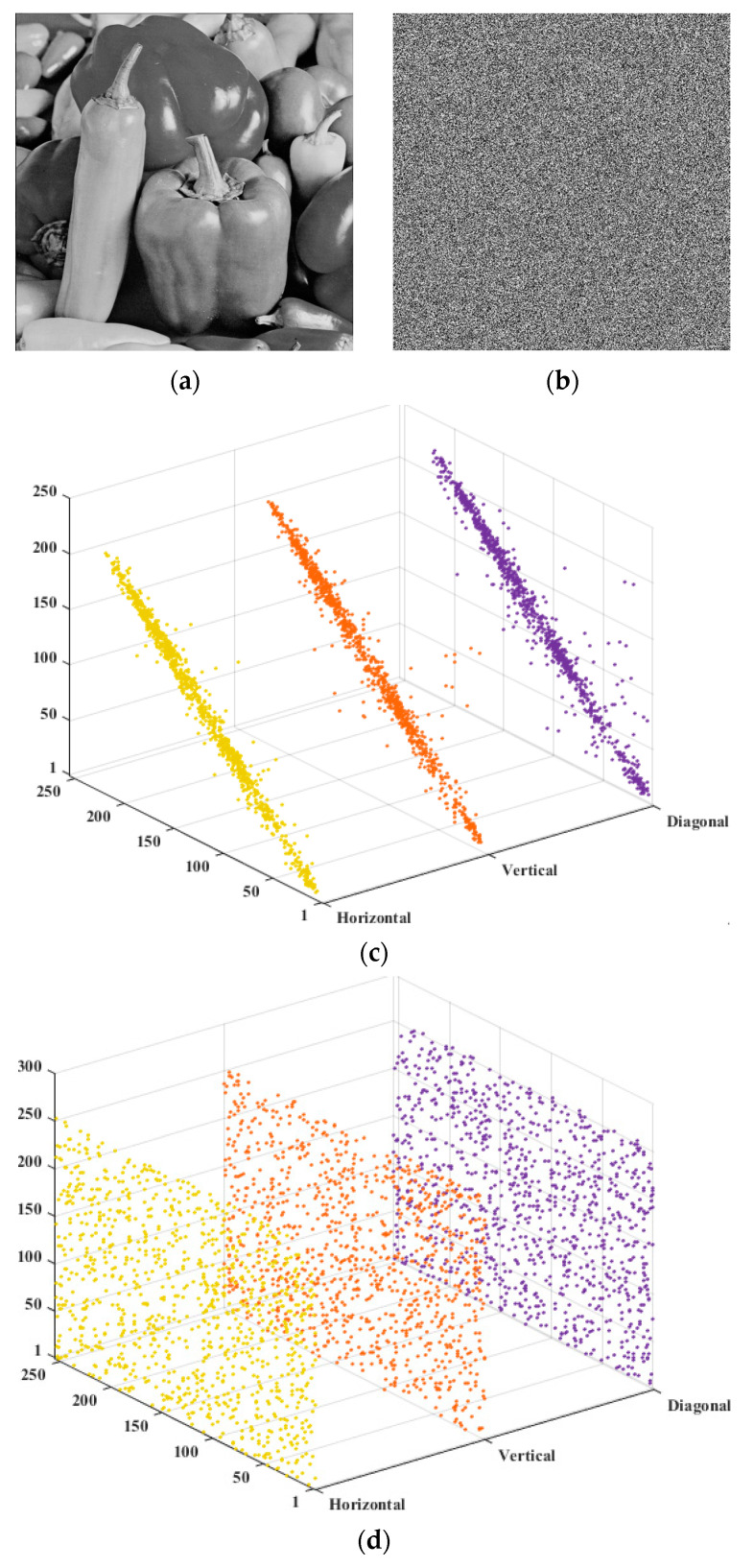
Correlation of adjacent pixels. (**a**) Plaintext image; (**b**) Ciphertext image; (**c**) The correlation coefficients of plaintext image; (**d**) The correlation coefficients of ciphertext images.

For plain images, the correlation coefficients are all above 0.9 and approach 1, indicating a very strong correlation among neighboring pixels. In contrast, the correlation coefficients of the encrypted images are close to 0, reflecting a weak or negligible correlation between adjacent pixels. Figure 14 visually shows the correlated intensities of adjacent pixels. The pixels of the plaintext image are distributed in linear proportions, while those of the encrypted image are distributed randomly, uniformly covering the entire coordinate plane. In addition, it can be seen from the data presented in Table 7 that the algorithm proposed in this paper has advantages over other algorithms, and that it is difficult for cryptographic images to provide valuable information for attackers. Therefore, our algorithm can achieve sufficient scrambling to protect the image information well.

#### 5.2.4. Information Entropy

The randomness of pixel intensity distribution within an image is commonly measured by information entropy. For an 8-bit grayscale image, information entropy is defined as follows:

(29) $$H(m)=\sum_{i=0}^{255}P(m_{i})\log_{2}\frac{1}{P(m_{i})},$$

where *m_i_* denotes the pixel value, and *P* (*m_i_*) is the occurrence probability of the gray value *m_i_*.

The information entropy measurements for the original and encrypted images, as well as those from alternative algorithms, are presented in Table 8. The entropy results for the ciphertext images are very near the theoretical maximum of 8. When compared to other algorithms, the proposed scheme exhibits superior performance.

#### 5.2.5. Local Shannon Entropy

To assess the randomness of the encrypted image from a localized standpoint, Local Shannon Entropy (LSE) is additionally employed as a qualitative metric. The LSE is defined as follows [39]:

(30) $$\bar{H_{t,t_{B}}}=\sum_{i=1}^{t}\frac{H(S_{i})}{t},$$

where *t* denotes the number of selected non-overlapping local blocks *S*_i_ within the image, and *t_B_* indicates the number of pixels contained in each block. For an 8-bit image, given a significance level of *a* = 0.001 with *t* = 30 and *t_B_* = 1936, the acceptable range for LSE is theoretically (7.8919, 7.9039) [42]. As reported in Table 8, the LSE values obtained for all encrypted images lie within this ideal interval.

### 5.3. Robustness Analyses

#### 5.3.1. Known Plaintext and Ciphertext Attacks

**Figure 15:**
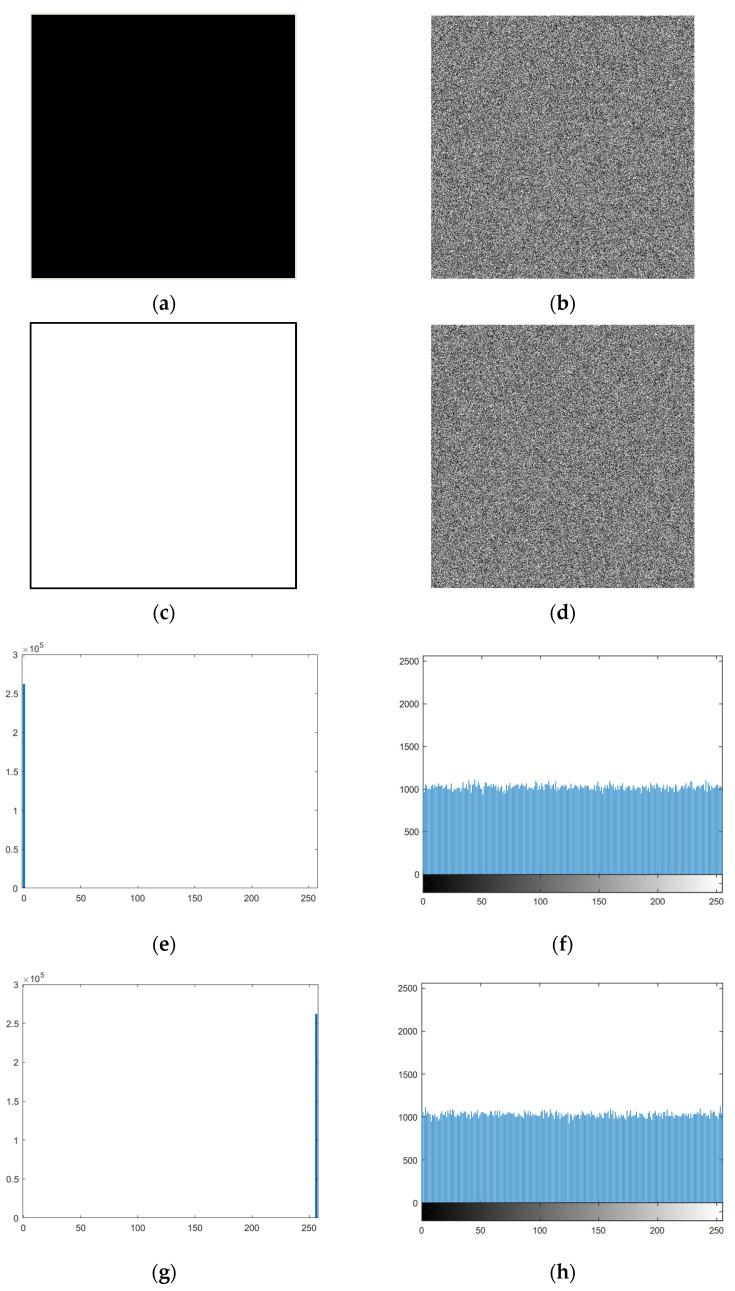
Experimental results for all-black and all-white images together with their histograms. (**a**) Black image; (**b**) Encrypted black image; (**c**) White image; (**d**) Encrypted white image; (**e**) Histogram of black image; (**f**) Histogram of encrypted black image (χ^2^ = 250.9551); (**g**) Histogram of white image; (**h**) Histogram of encrypted white image (χ^2^ = 236.7793).

In our algorithm, the key stream is correlated with the plaintext by the SHA-256 hash algorithm. Simultaneous scrambling–diffusion operations have sufficiently altered the plain image, and the proposed algorithm is unaffected by the known plaintext attack. Figure 15a–d show the ‘Black’, the ‘White’ and their corresponding cipher images, and Figure 15e–h are their histograms. Both the chaotic encrypted images and uniform histograms all show that the algorithm can effectively resist the known plaintext attacks.

We conducted the evaluation of this algorithm’s resistance to chosen-plaintext attacks through systematic differential attack testing. During the testing process, we constructed plaintext image pairs (*I*_1_, *I*_2_) with single-pixel differences and encrypted them using identical keys to obtain corresponding ciphertext pairs (*E*_1_, *E*_2_). The diffusion characteristics of the algorithm were quantitatively analyzed by calculating two key metrics: the NPCR and UACI. Experimental results demonstrate that for standard 512 × 512 test images, the proposed algorithm achieves an average NPCR of 99.61% (theoretical ideal value: 99.6094%) and an average UACI of 33.47% (theoretical ideal value: 33.4635%) [41], with deviations from theoretical values not exceeding 0.003%. These results significantly outperform those reported in comparative studies.

#### 5.3.2. Noise Attack

**Figure 16:**
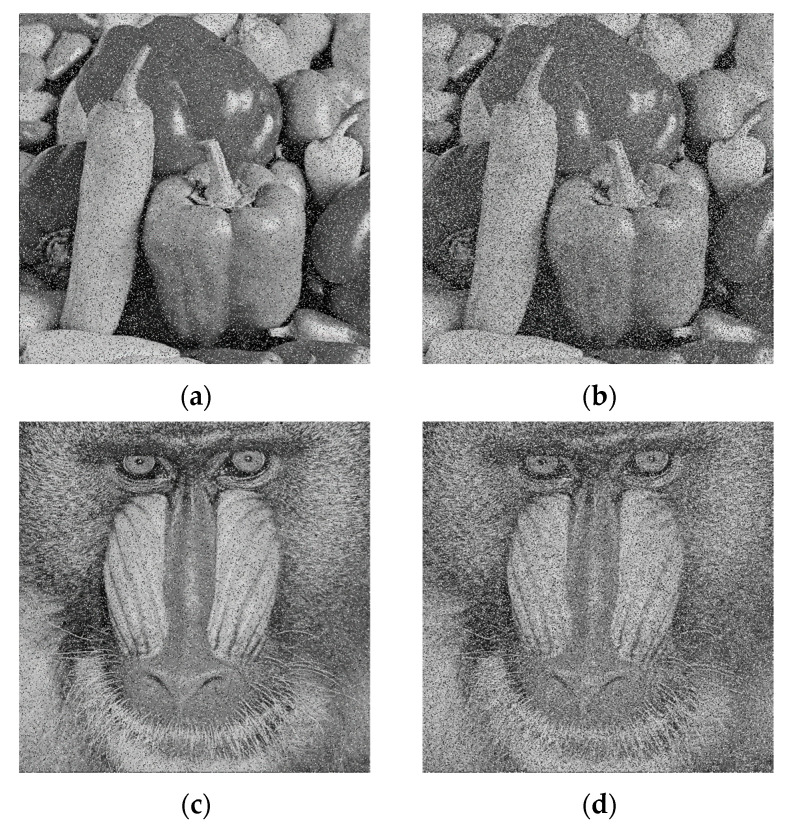
Analysis of Salt-and-Pepper noise effects. (**a**) Peppers image with noise density of 0.05; (**b**) Peppers image with noise density of 0.1; (**c**) Baboon image with noise density of 0.05; (**d**) Baboon image with noise density of 0.1.

**Figure 17:**
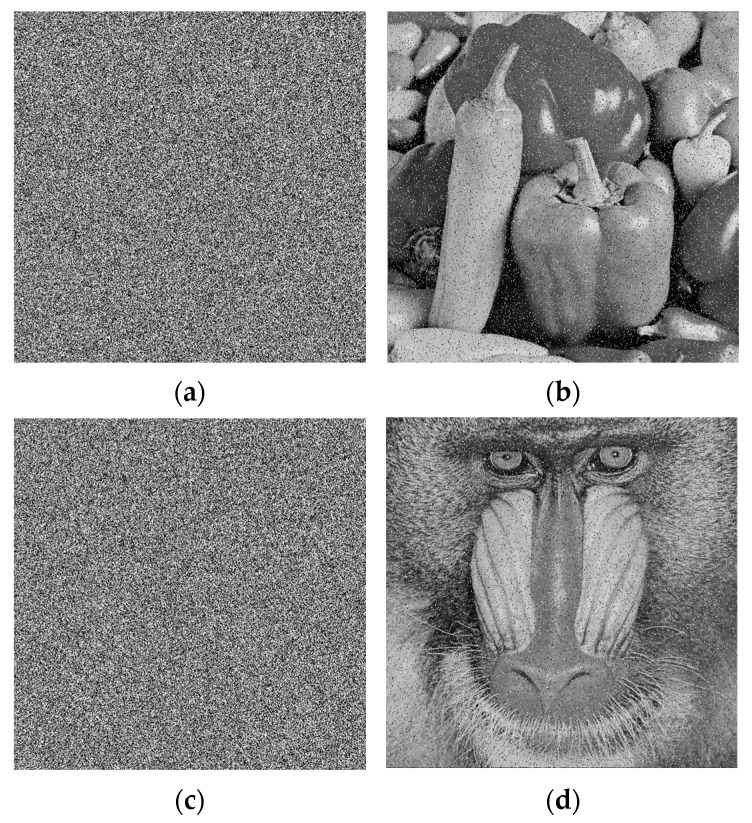
Analysis of impulse noise effects. (**a**) Encrypted noisy Peppers; (**b**) Decrypted Peppers image; (**c**) Encrypted noisy Baboon; (**d**) Decrypted Baboon image.

The encrypted Peppers and Baboon images were superimposed with salt-and-pepper noise at different intensities, i.e., 0.05 and 0.1 The decrypted image is shown in Figure 16. Figure 17 shows the encrypted image with noise and the decrypted image, respectively.

The experimental results demonstrate that the proposed algorithm is effective against both salt-and-pepper noise and impulse noise attacks. For images that have been degraded by noise, the Peak Signal-to-Noise Ratio (PSNR) serves as a crucial metric for assessing image quality. A higher PSNR value indicates less distortion and clearer image content. The definitions of the Mean Square Error (MSE) and PSNR are given as follows:

(31) $$MSE=\frac{1}{m\times n}\sum_{i=1}^{m}\sum_{j=1}^{n}{\left[P(i,j)-C(i,j)\right]}^{2},$$

(32) $$PSNR=10\times \log_{10}\left[\frac{{\left(2^{b}-1\right)}^{2}}{MSE}\right],$$

where *P* denotes the original plaintext image, *C* represents the decrypted image, and *b* is the number of bits used to represent each pixel. The resulting PSNR values are reported in Table 9.

#### 5.3.3. Cropping Attack

**Figure 18:**
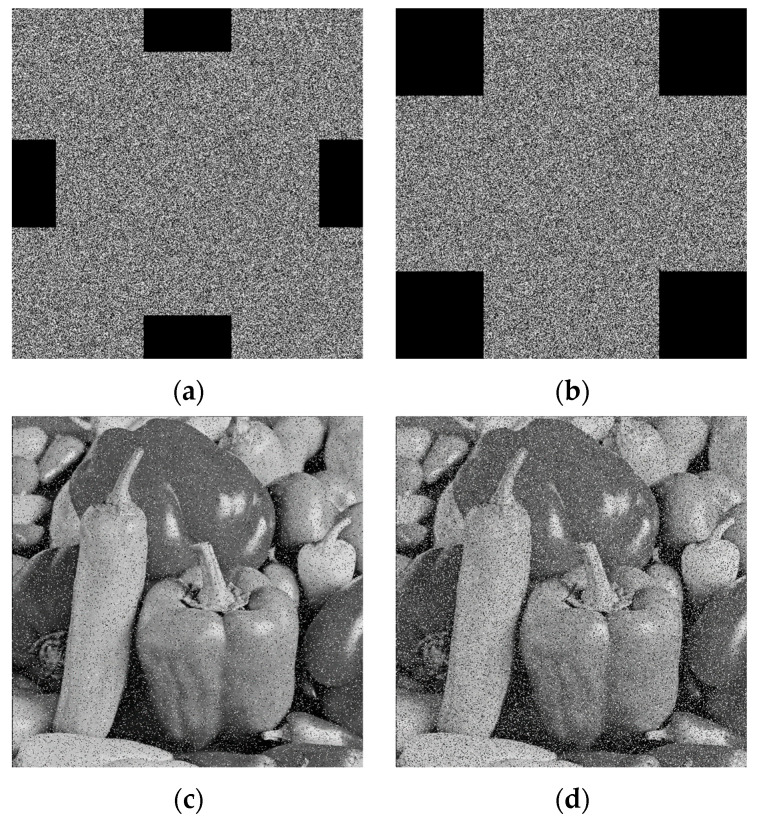
Performance under clipping attacks for the Peppers image. (**a**) Cropping of 1/8 of the image; (**b**) Cropping of 1/4 of the image; (**c**) Decryption of (**a**); (**d**) Decryption of (**b**).

**Figure 19:**
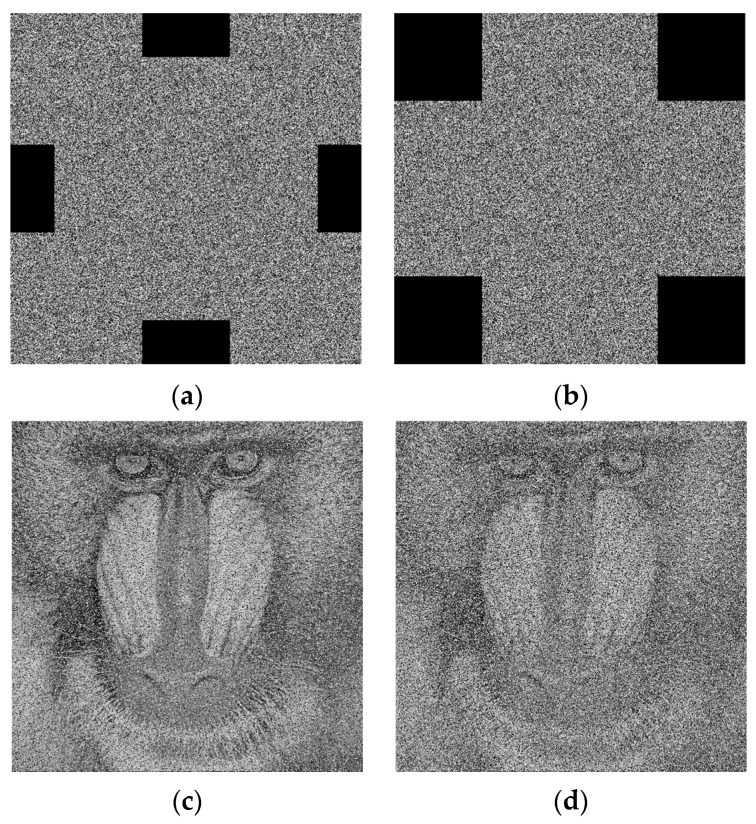
Performance under clipping attacks for the Baboon image. (**a**) Cropping of 1/8 of the image; (**b**) Cropping of 1/4 of the image; (**c**) Decryption of (**a**); (**d**) Decryption of (**b**).

Clipping attacks simulate the loss of information that may be experienced during channel transmission. Resistance to this is verified by testing the encrypted images with different cropping positions or cropping sizes. The decrypted results of the Peppers and Baboon images are shown in Figure 18 and Figure 19, respectively. The corresponding PSNR values are shown in Table 10. Obviously, with the increase in the cropping area, the decrypted images still contain the main information, although the quality is reduced. Therefore, the algorithm is robust to clipping attacks.

### 5.4. Efficiency Analyses and Time Complexity

For encryption algorithms, speed is as important as security. Table 11 gives a list of the average encryption time and makes comparisons with the speed of other similar algorithms. It shows that a 512 × 512 image takes 0.7 s on average to encrypt with the proposed algorithm. Therefore, our algorithm is efficient.

Through theoretical analysis, we evaluate the computational complexity of the proposed algorithm. In the preprocessing phase, both image reading and SHA-256 hash computation exhibit linear time complexity O(*mn*), where m and n denote the image height and width, respectively. Chaotic sequence generation employs an improved 2D-CACCM system requiring 1000 + 2mn iterations, maintaining O(*mn*) complexity.

Sorting *mn* chaotic elements contributes O(*mn*log(*mn*)) complexity, constituting the dominant factor in this phase. The extension of the 2D image to a 3D *m* × *n* × 256 matrix involves traversing each pixel once to populate its spatial position, resulting in O(*mn*) complexity. The 3D simultaneous scrambling–diffusion step traverses the 3D matrix, applying permutation and diffusion via precomputed index sequences, which preserves O(*mn*) complexity since each pixel is processed once. The 2D XOR diffusion has the same complexity of O(*mn*). Finally, merging the 3D matrix into a 2D encrypted image by summing values along the z-axis per pixel is also an O(*mn*) operation. The overall time complexity is dominated by the sorting operation during chaotic-sequence processing, yielding O(*mn*). This efficiency demonstrates the algorithm’s suitability for real-time image-transmission applications.

## 6. Conclusions

To improve the security of encryption, an image 3D encryption algorithm based on a three-dimensional histogram model is proposed. The algorithm adopts the structure of simultaneous scrambling–diffusion. On the one hand, a chaos-based 3D simultaneous scrambling–diffusion system is designed to change pixel values while scrambling with an index. On the other hand, 2D XOR diffusion can achieve further diffusion. The proposed algorithm has a sufficiently large key space and good key sensitivity and can resist brute-force attacks such as enumeration attacks. The encrypted images have uniform histograms and pass the Chi-square test. The information entropy is greater than 7.9992, and the local information entropy is also within the theoretical range. The average values of NPCR and UACI show that this algorithm can effectively resist differential attacks. The pixels of an encrypted image are almost completely uncorrelated. Noise and pruning attacks show that the algorithm has good robustness. Finally, through the analysis of encryption time, it is proven that the algorithm can be applied to real-time image transmission.

## Figures and Tables

**Table 1:** NIST test results of the 2D-CACCM.

Statistical Tests	*p*-Value	Results
Frequency	0.149302	Passed
Block Frequency	0.245954	Passed
Runs	0.668592	Passed
Longest Run	0.496398	Passed
Rank	0.040769	Passed
FFT	0.601431	Passed
Non-overlapping Template	0.209521	Passed
Overlapping Template	0.092170	Passed
Universal	0.373174	Passed
Linear Complexity	0.612013	Passed
Serial test *p*-value 1	0.155170	Passed
Serial test *p*-value 2	0.343248	Passed
Approximate Entropy	0.063794	Passed
Cumulative Sums-forward	0.814758	Passed
Cumulative Sums-reverse	0.156815	Passed
Random Excursions Test (X = 1)	0.072391	Passed
Random Excursions Variant Test (X = 1)	0.022622	Passed

**Table 2:** Key space comparisons.

Algorithm	Proposed	Ref. [37]	Ref. [38]	Ref. [39]	Ref. [40]	Ref. [41]
Key space	2^199^	2^154^	10^42^	2^200^	2^384^	10^108^

**Table 3:** Analysis of key sensitivity.

Figure	Decryption Key	Pixel Difference Ratio
Figure 12a	*key*_0_ = {*a*, *b*, *x*_0_, *y*_0_}	0.0%
Figure 12b	*key*_1_ = {*a* + 10^−14^, *b*, *x*_0_, *y*_0_}	99.6330%
Figure 12c	*key*_2_ = {*a*, *b*, *x*_0_ + 10^−14^, *y*_0_}	99.5987%
Figure 12d	*key*_3_ = {*a*, *b*, *x*_0_, *y*_0_ + 10^−14^}	99.6075%

**Table 4:** Analysis of differential attack (PSNR and UACI).

Image Size	Image Name	NPCR	UACI
256 × 256	5.1.09	99.6094	33.4819
	5.1.10	99.6017	33.4696
	5.1.11	99.6182	33.4564
	5.1.12	99.6439	33.4867
	5.1.13	99.5955	33.4968
512 × 512	Baboon	99.5987	33.4755
	Boat	99.6014	33.4555
	House	99.6155	33.4660
	Peppers	99.6443	33.4593
	Tank	99.6424	33.4669
	Ruler	99.5940	33.4765
1024 × 1024	1.3.03	99.6161	33.4817
	1.4.03	99.6244	33.4775
	1.4.05	99.6172	33.5026
	3.2.25	99.6329	33.4811

**Table 5:** NPCR and UACI values of the images under different algorithms.

Image Size	Index	NPCR	UACI
256 × 256	Proposed (on average)	99.6137	33.4783
	Ref. [34]	99.6312	33.4531
	Ref. [39]	99.6078	33.5708
	Ref. [40]	99.5911	33.4614
512 × 512	Proposed (on average)	99.6160	33.4666
	Ref. [34]	99.6162	33.5251
	Ref. [37]	99.6080	33.4720
	Ref. [38]	99.5911	33.6038
	Ref. [39]	99.6017	33.4556
	Ref. [40]	99.6025	33.5151
	Ref. [44]	99.6185	33.4533
	Ref. [45]	99.6470	28.5198
1024 × 1024	Proposed (on average)	99.6226	33.4857
	Ref. [40]	99.6127	33.5031
	Ref. [44]	99.6102	33.4632
	Ref. [45]	99.5278	32.6692

**Table 6:** Test results of Chi-squared tests.

Image	χ^2^-Value	1% Probability		5% Probability	
Peppers	267.3145	310.4570	Passed	293.2478	Passed
Baboon	263.8730		Passed		Passed
Boat	275.3438		Passed		Passed
House	243.8613		Passed		Passed
Tank	267.2441		Passed		Passed
Ruler	281.8184		Passed		Passed

**Table 7:** Adjacent pixel correlation results for multiple images.

Plain Image	Test Image	Horizontal	Vertical	Diagonal
Peppers	Original image	0.9794	0.9767	0.9634
	Encrypted image	0.0035	−0.0108	−0.0056
Baboon	Original image	0.7524	0.8607	0.7148
	Encrypted image	0.0034	−0.0061	0.0041
Boat	Original image	0.9720	0.9402	0.9233
	Encrypted image	0.0033	0.0115	−0.0016
House	Original image	0.9546	0.9519	0.9153
	Encrypted image	−0.0084	0.0029	−0.0186
Lena	Original image	0.9862	0.9742	0.9584
	Encrypted image	0.0012	−0.0047	0.0062
Peppers [37]	Encrypted image	0.0116	−0.0158	−0.0055
Boat [37]	Encrypted image	0.0064	0.0076	0.0159
Peppers [38]	Encrypted image	−0.0021	−0.0072	0.0003
Baboon [38]	Encrypted image	−0.0176	−0.0067	−0.0151
Lena [41]	Encrypted image	0.0182	0.0213	−0.0160
Peppers [40]	Encrypted image	0.0064	−0.0110	−0.0088

**Table 8:** Entropy analysis (global and local) for multiple images.

Image	Plain Image	Encrypted Image	LSE	Result
Peppers	7.5937	7.9993	7.9006	Passed
Baboon	7.3723	7.9993	7.9015	Passed
Boat	7.1914	7.9992	7.9017	Passed
House	7.2334	7.9993	7.9036	Passed
Tank	5.4957	7.9993	7.9002	Passed
Lena	7.4472	7.9994	7.9028	Passed
Average	7.0556	7.9993	7.9017	Passed
Boat [37]	7.1913	7.9993	-	-
Peppers [37]	7.5251	7.9971	-	-
Peppers [38]	7.5797	7.9971	7.8993	Passed
Baboon [38]	7.0092	7.9970	7.9034	Passed
Hand [41]	7.7453	7.9988	-	-

**Table 9:** Objective quality assessment (PSNR and MSE) of noisy images.

Noise Type	Noise Intensity	Peppers		Baboon	
		PSNR	MSE	PSNR	MSE
Salt and Pepper noise	0.05	16.1495	1578	16.8312	1348
	0.1	13.5928	2843	14.1677	721
Impulse noise	0.1	18.8282	851	19.5513	721

**Table 10:** Evaluation of cropped images using PSNR.

Image	1/8 Clipping	1/4 Clipping
Peppers	8.6668	7.6522
Baboon	8.9329	8.0049

**Table 11:** Time required for encryption and decryption processes.

Algorithm	Image	Encryption Time (s)	Decryption Time (s)
Proposed	Peppers	0.7047	0.9204
	Baboon	0.7228	0.9344
	Boat	0.7024	1.0020
	House	0.6862	0.9891
	Ruler	0.7122	0.9703
	Average	0.7056	0.9632
Ref. [23]	Cameraman	0.8970	1.0220
Ref. [34]	-	0.8408	-
Ref. [44]	-	0.5118	-
Ref. [42]	Lena	0.4624	0.2698

## Data Availability

The original contributions presented in this study are included in the article. Further inquiries can be directed to the corresponding author.
